# Effect of a single nonpharmacological intervention on cognitive functioning in older adults with mild-to-moderate Alzheimer's disease: A meta-analysis of randomized controlled trials

**DOI:** 10.1016/j.tjpad.2024.100050

**Published:** 2025-01-01

**Authors:** Kejin Chen, Xiaoyan Zhao, Jingwen Zhou

**Affiliations:** a Changzhou maternal and Child Health Care Hospital, Changzhou Medical Center, Nanjing Medical University, Changzhou 213000, Jiangsu, China; b Medical Innovation Research Department, Chinese People's Liberation Army General Hospital, Beijing 100000, China; c Hospital-Acquired Infection Control Department, Affiliated Nanjing Brain Hospital, Nanjing Medical University, Nanjing 210000, Jiangsu, China

**Keywords:** Non-medical prescribing, Cognition, Alzheimer disease, Aged, Meta-analysis

## Abstract

Most studies of nonpharmacological interventions have used a combination of medications in experimental and control groups to improve cognitive functioning or to control symptoms, but the results have been inconsistent with respect to the effects of single nonpharmacological interventions on cognitive functioning in older patients with Alzheimer's disease. The aim of this study was to assess the effect of a single nonpharmacological intervention on cognitive functioning in older adults with mild-to-moderate Alzheimer's disease. We conducted a systematic review and meta-analysis in the first week of January 2024, searching eight electronic databases for articles that reflect on non-pharmacological interventions in Alzheimer's disease published between January 1, 1986, and December 31, 2023. All included articles had to be randomized controlled trials. The primary measure was the change in cognitive function before and after the intervention. Data were extracted by two authors and quality was assessed using the Cochrane Handbook. With the exception of the Montreal Cognitive Assessment (MoCA) scale [MD=2.99, 95% CI (-0.66,6.63)], the differences between the intervention group and the control group were significant for all the remaining scales, namely, the Mini-Mental State Examination (MMSE) [SMD=0.65, 95% CI (0.15,1.15)], Activity of Daily Living Scale (ADL) [MD=-2.30, 95% CI (-3.63,0.97)], Quality of Life in Alzheimer's Disease Scale (QoL-AD) [MD=5.03, 95% CI (2.27,7.78)], Neuropsychiatric Inventory (NPI) [MD=-2.16, 95% CI (-3.86,0.46)], and Alzheimer's Disease Assessment Scale-cognitive score (ADAS-cog) [MD=-5.21, 95% CI (-7.89,2.54)]. Subgroup analysis revealed that the most effective intervention was exercise therapy, followed by repetitive transcranial magnetic stimulation. On the other hand, music therapy was not found to be effective. Current evidence suggests that nonpharmacological interventions can be used to improve cognitive functioning in older adults with mild-to-moderate Alzheimer's disease. This study was registered in PROSPERO (registration number: CRD42024497247).

## Introduction

Alzheimer's disease (AD) is a disease characterized by a transition from health and independence to a state of dependence and a progressive loss of memory and functional skills [1]. AD patients experience impairments in several cognitive domains. According to the 2015 World Alzheimer Report [2], there were approximately 50 million people with dementia worldwide in 2015, and it is expected that by 2050, there will be 132 million people with dementia, with Alzheimer's disease accounting for approximately 63% to 70% of dementia cases. The most recent data suggest that the incidence of dementia will double in Europe and triple globally by 2050 [3]. The prevalence of Alzheimer's disease in China is increasing – a national cross-sectional study published in December 2020 showed that among adults aged 60 years and older in China, there were 15.07 million people with dementia and 9.83 million people with Alzheimer's disease, thus yielding a 3.9% prevalence rate of AD after correcting for age and sex [4]. Some studies have predicted that without effective preventive measures, the number of Alzheimer's disease patients among Chinese people aged 60 and above will increase substantially in the next 30 years, reaching 20.75 million in 2030 and up to 30.03 million in 2050 [5].

Between 2000 and 2013, the number of deaths from Alzheimer's disease in the United States increased by 71%; accordingly, Alzheimer's disease was the sixth leading cause of death in the United States in 2013 and the fifth leading cause of death among Americans aged ≥65 years [6]. These numbers are derived from official death certificates, and the actual number of deaths caused by Alzheimer's disease may be far greater. Alzheimer's disease imposes a heavy economic burden on society and families, with the per capita annual cost of AD patients in China amounting to $19,144.36 in 2015, and the total socioeconomic burden due to AD reaching $167.74 billion [7].

Despite the fact that Alzheimer's disease is a major public health problem, only five treatments are currently approved to control symptoms rather than alter the course of the disease [8]. Cholinesterase inhibitors and memantine are approved treatments, but they only control symptoms and do not change the course of the disease. Multidomain lifestyle-based prevention trials have shown that participants at increased risk for dementia benefit cognitively from these treatments. Lifestyle factors do not directly influence the pathology of Alzheimer's disease but can still lead to positive outcomes for people with Alzheimer's disease [3]. For example, research has shown that arithmetic, drawing, and writing are effective in improving communication skills and quality of life [9]. Furthermore, underlying medical conditions can sometimes lead to misdiagnosis or the overprescribing of psychiatric medications in individuals who only present with mental symptoms but cannot be diagnosed with a psychiatric disorder [10]. Implementing nonpharmacological interventions might help improve this situation.

Alzheimer's disease has been a hot topic of research in recent years. However, there are few empirical studies on the use of nonpharmacological interventions to treat and prevent AD. Nonpharmacological interventions for AD refer to treatments that professionals can offer without the use of synthetic drugs, thereby avoiding side effects. Examples include widely popular therapies such as psychotherapy and exercise therapy, as well as transcranial brain stimulation methods that have gained widespread use in recent years. Transcranial brain stimulation methods, such as transcranial magnetic (TMS) and transcranial electric (tES) stimulation, the latter is further divided into transcranial direct current stimulation (tDCS) and transcranial alternating current stimulation (tACS), have been extensively used in an effort to improve cognitive functions in humans [11]. The vast majority of studies on nonpharmacological interventions have used medications to improve cognitive functioning or control symptoms throughout both the test and control groups. Although stable dosages were maintained in these studies, there was a lack of strong evidence for the interpretation of study conclusions. For example, Cristina Fonte's team [12] confirmed the positive effects of cognitive and physical activity treatments in terms of mitigating cognitive decline in AD patients; however, participants were on medication during the study, and the number of people in each group varied. This may have affected the reliability of the conclusions, and there have been numerous studies with similar limitations [[13], [14], [15]]. The previous reviews of non-pharmacological interventions focus on lifestyle modifications and nutritional interventions [16,17], suggesting that both may have potential benefits for cognitive decline and dementia. In recent years, meta-analyses have also studied Alzheimer's disease populations specifically through transcranial magnetic stimulation (TMS) [18].

Moreover, findings on the effects of purely nonpharmacological interventions on cognitive functioning in older adults with Alzheimer's disease have been inconsistent. For example, rTMS therapy has been found to be effective for improving cognitive functioning in AD patients some studies [19,20] but not in other studies [21,22]. In addition, some studies of nonpharmacological interventions combined multiple interventions, and the final conclusions were generated by the interaction of multiple methods [[23], [24], [25]]; therefore, it was not possible to assess the impact of a single nonpharmacological intervention.

It is necessary to conduct more high-quality studies on this topic. Therefore, this study aimed to investigate the effects of single nonpharmacological interventions on cognitive functioning in older adults with mild-to-moderate Alzheimer's disease. The results of this meta-analysis may inform health care providers and researchers regarding the effectiveness and magnitude of the impact of nonpharmacological interventions to improve cognitive functioning in older adults with mild-to-moderate Alzheimer's disease. Furthermore, the findings should provide implications for clinical practice and future research. Moreover, the results will inform the choice of nonpharmacological interventions for direct caregivers of older patients.

## Materials and methods

### Search strategy

The following databases were systematically searched in the first week of January 2024: Juhe Biomedical Knowledge Service Platform (JKS-MED), WANFANGMED ONLINE, Chinese Biomedical Literature Database (CBM), China National Knowledge Infrastructure (CNKI) Database, Cochrane Library, Web of Science (WOS), Excerpta Medica Database (EMBase), and PubMed. The languages were limited to Chinese or English. There was no restriction regarding publication date.

**Fig. 1:**
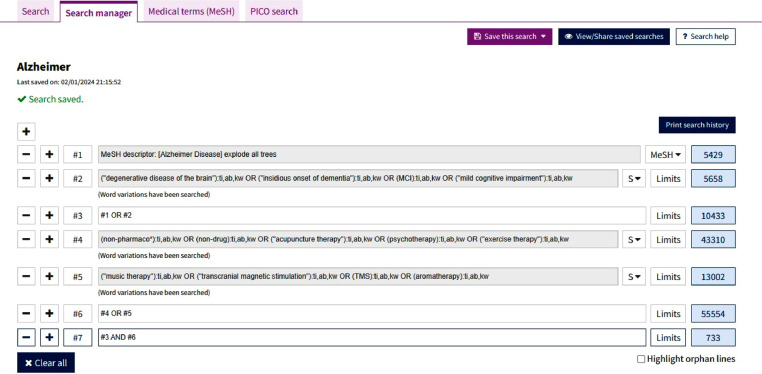
The search strategy and results of Cochrane Library.

Medical Subject Heading (MeSH) terms, keywords, and free words were used to search for potentially eligible studies, such as "Alzheimer Disease", "mild cognitive impairment", "TMS", and "exercise therapy". The proposed keywords and combinations are shown in Table 1. The specific search strategy and results for the Cochrane Library are shown in Fig. 1.

**Table 1:** Proposed keywords and combinations.

Population		Intervention
Alzheimer Disease	**AND**	non-pharmaco*
**OR**		**OR**
"degenerative disease of the brain"		non-drug
**OR**		**OR**
"insidious onset of dementia"		"acupuncture therapy"
**OR**		**OR**
MCI		psychotherapy
**OR**		**OR**
"mild cognitive impairment"		"exercise therapy"
		**OR**
		"music therapy"
		**OR**
		"transcranial magnetic stimulation"
		**OR**
		TMS
		**OR**
		aromatherapy

### Selection criteria

The single non-pharmacological intervention indicates that there are specific requirements regarding the quantity and type of intervention methods involved, referring to the usage of only one non-pharmacological intervention method. This means that combinations of several non-pharmacological methods or the inclusion of pharmacological methods are not permitted.

The inclusion criteria were as follows: randomized controlled trials regardless of blinding methods; the target population was older adults with mild-to-moderate Alzheimer's disease; outcome scales assessed changes in cognitive functioning; and studies published in a peer-reviewed journal in Chinese or English. The exclusion criteria were as follows: the target population mentioned only mild cognitive impairment; for duplicate published studies, we selected the article with the most comprehensive data; drugs to improve cognitive function, such as cholinesterase inhibitors, memantine, antipsychotics, and sedative-hypnotics, were not given to both the test and control groups during the intervention; studies where data could not be extracted accurately or were missing; studies involving combinations of multiple nonpharmacological interventions; and clinical trials that were published in the pre-registration phase. Two researchers independently screened and cross-checked the literature. Disagreements were resolved through discussion or by consulting a third researcher. For studies with incomplete data, we reached out to the corresponding authors via email to request the missing data.

### Outcome measures

The outcome of interest was the change in scores on cognitive functioning scales and its standard deviation. We screened the literature by considering the inclusion of the following six internationally recognized cognitive functioning scales: the Mini-Mental State Examination (MMSE), Activity of Daily Living Scale (ADL), Quality of Life in Alzheimer's Disease (QOL-AD) scale, Montreal Cognitive Assessment (MoCA), Neuropsychiatric Inventory (NPI), and Alzheimer's Disease Assessment Scale-cognitive Scale (ADAS-cog). Since the scale scores were continuous variables, the standardized mean difference (SMD) or difference in means (MD) was used to indicate the difference in each scale score between the nonpharmacological intervention group and the control group.

### Data extraction

The relevant information was extracted and verified by two independent evaluators. Differences were resolved through discussion or by consulting a third researcher. The following data were extracted: basic information, including the first author, year of publication, and country; basic characteristics of the research subjects, including sample size, age, and disease duration; detailed information on the duration and frequency of intervention; intervention effectiveness indicators, such as the means and standard deviations of the pretests and posttests; and differences between these two scales for the intervention and control groups.

### Quality assessment

The risk of bias among the included studies was evaluated using the RCT bias risk assessment tool recommended in version 5.1.0 of the Cochrane Handbook. The following domains of risk of bias were evaluated: whether the randomization method was correct; whether the allocation was concealed; whether the subjects and researchers were blinded; the completeness of the outcome data; selective reporting of research results; and other sources of bias. The risk of bias was independently evaluated by two reviewers, and the results were cross-checked. Differences were resolved through discussion.

### Statistical analysis

Meta-analysis was performed with RevMan 5.3 and STATA 17.0 software. The SMD or MD was used as the statistic for effect analyses, and 95% confidence intervals (CIs) were calculated. The heterogeneity of the included studies was assessed using the χ2 test (the test level was α= 0.1) and the *I^2^* statistic. In cases of considerable heterogeneity, the random effects model was used for analysis, or subgroup analysis was performed to identify sources of heterogeneity. Publication bias was judged by constructing a clipping-contour-enhanced funnel plot.

## Results

### Data sources

A total of 12,792 citations were initially retrievd. We used filters to screen out randomized controlled trials among the English databases.

**Fig. 2:**
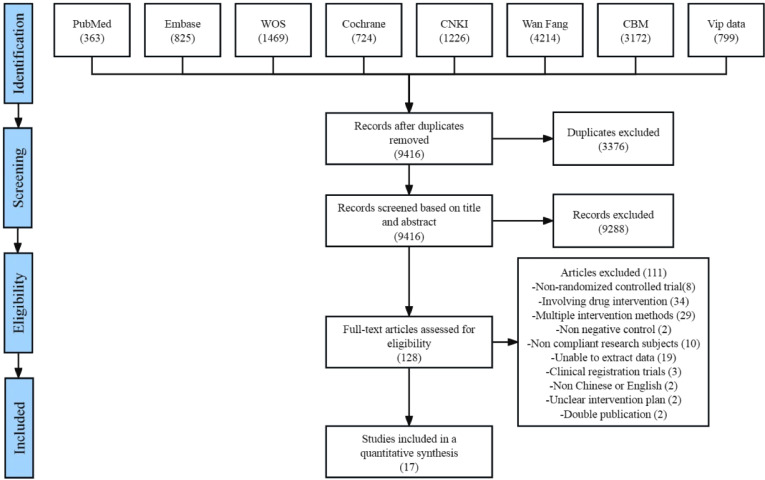
Literature search and study selection.

After removing duplicate entries, 9416 articles remained. The titles and abstracts of these articles were screened, and 9288 papers that did not meet the inclusion criteria were excluded. The remaining 128 papers were read in full for further screening. Seventeen original studies were ultimately included in the meta-analysis [[19], [20], [21], [22],[26], [27], [28], [29], [30], [31], [32], [33], [34], [35], [36], [37], [38]], as shown in Fig. 2.

### Basic characteristics of the included studies

The basic information of the included studies, the basic characteristics of the research subjects, and the detailed information of the intervention measures are summarized in Table 2.

**Table 2:** Characteristics and outcomes of a nonpharmacological intervention for older adults with mild-to-moderate Alzheimer's disease.

### Mini-mental state examination

**Fig. 3:**
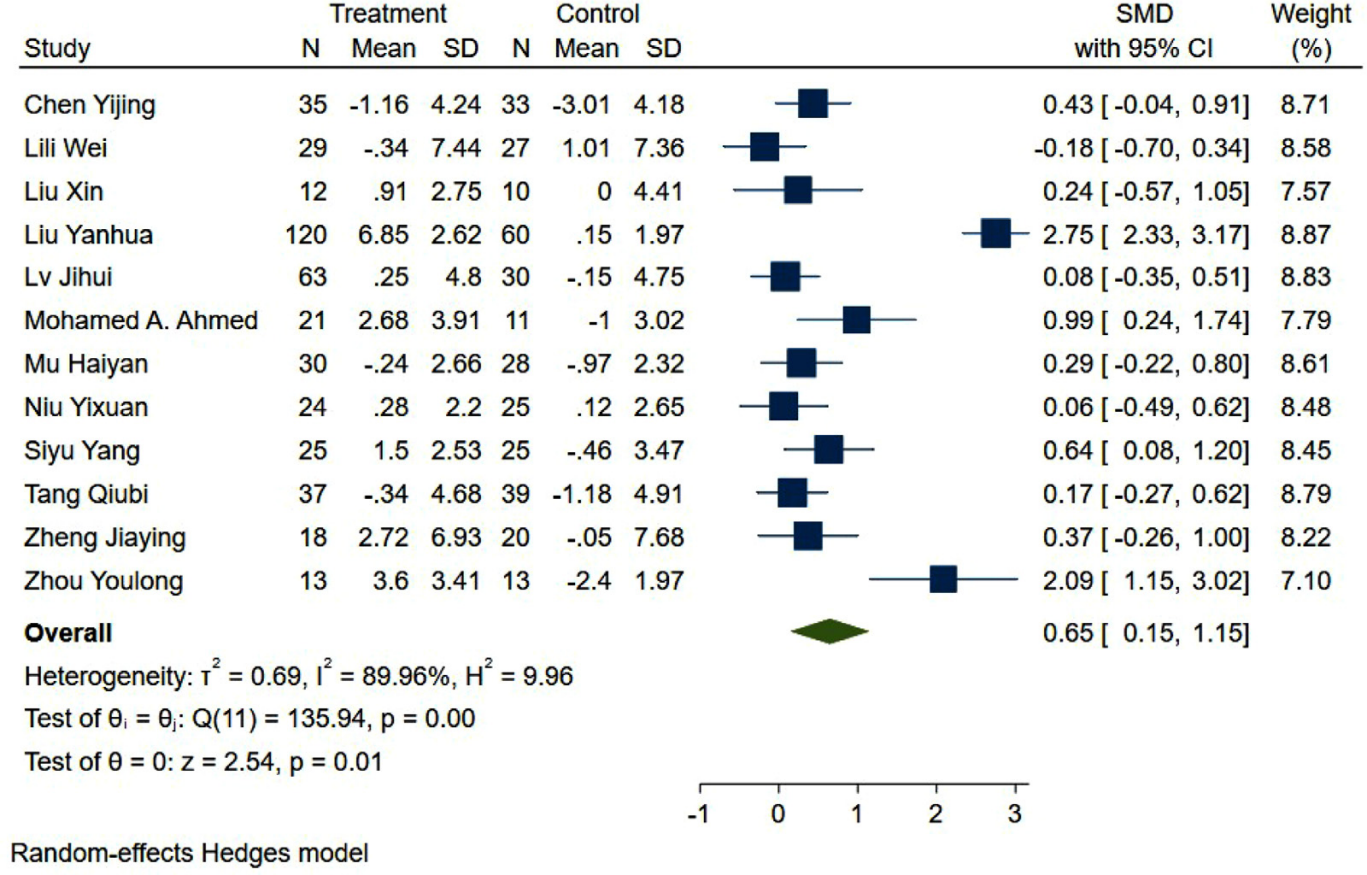
Forest plot of MMSE scores in older adults with Alzheimer's disease in the nonpharmacological intervention group versus control group.

A total of 427 older adults with Alzheimer's disease who underwent nonpharmacological interventions and 321 who did not undergo nonpharmacological interventions completed the MMSE before and after treatment. The random effects model was used for meta-analysis, and the results showed that the mental state of the nonpharmacological intervention group was better than that of the control group [SMD=0.65, 95% CI (0.15,1.15)] (Fig. 3). There was significant heterogeneity (*I^2^* =89.96%, *P* = 0.01).

**Fig. 4:**
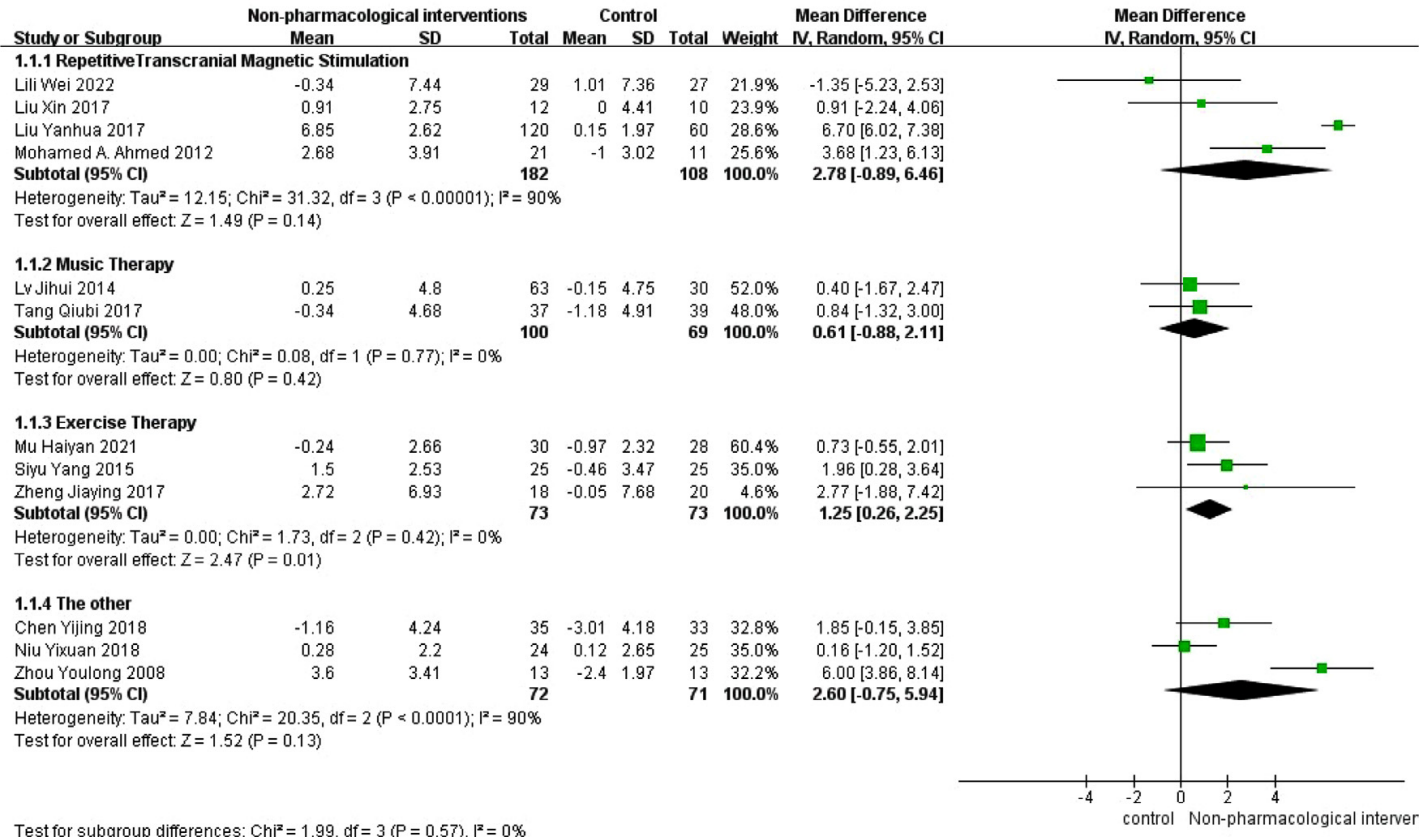
Results of subgroup analysis of MMSE scale scores.

To address the high level of heterogeneity, we conducted subgroup analyses based on the various interventions. The heterogeneity may have been due to the use of repetitive transcranial magnetic stimulation (rTMS) and other treatments. The results of the subgroup analysis showed that only the exercise group had significantly different MMSE scores from those of the control group (Fig. 4).

### Activity of daily living scale

**Fig. 5:**
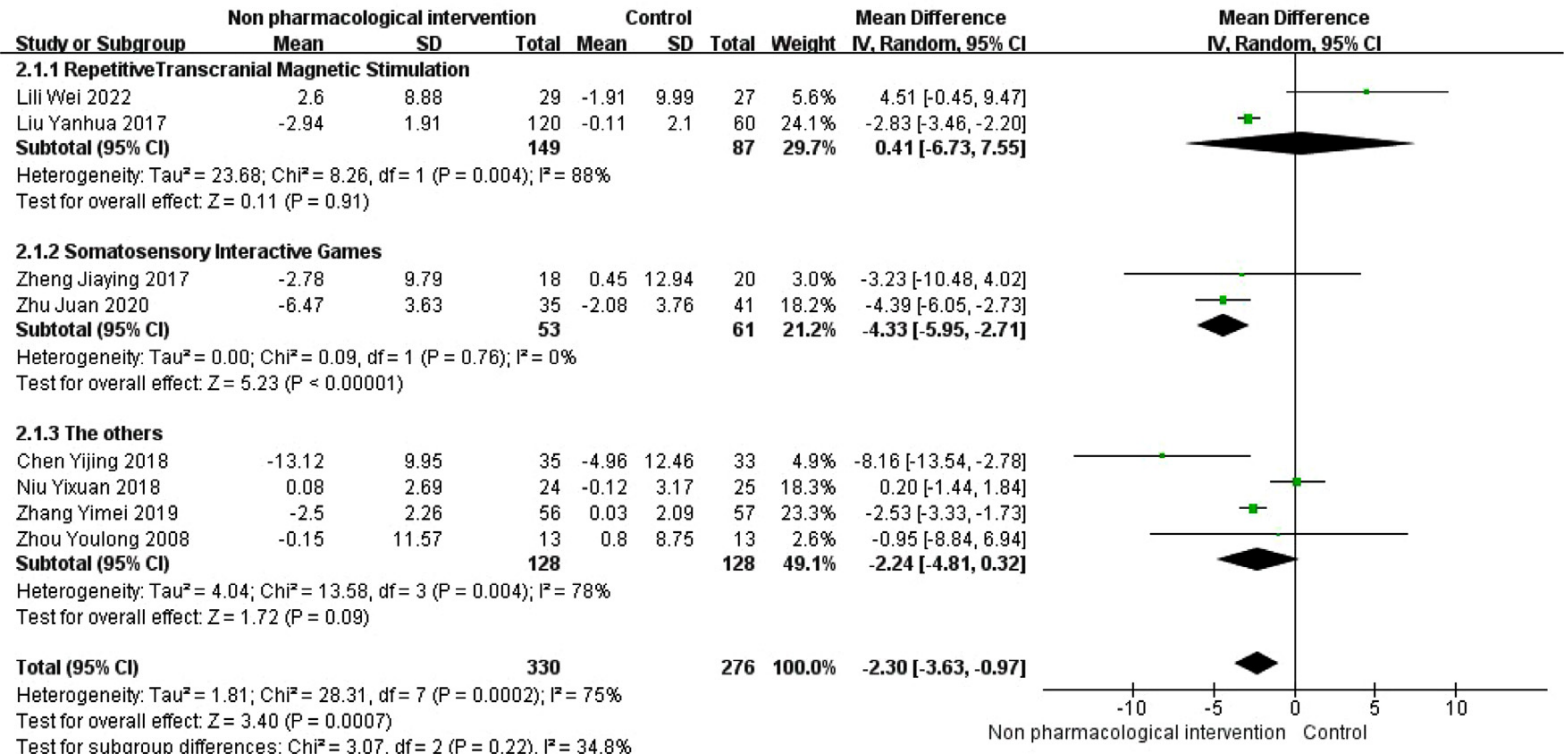
Forest plot of ADL scores in older adults with Alzheimer's disease in the nonpharmacological intervention group versus control group.

A total of 330 older adults with Alzheimer's disease who underwent nonpharmacological interventions and 276 who did not undergo nonpharmacological interventions completed the ADL scale before and after treatment. The random effects model was used for meta-analysis, and the results showed that the daily living skills of the nonpharmacological intervention group were better than those of the control group [MD=−2.30, 95% CI (−3.63,0.97)] (Fig. 5). There was significant heterogeneity (*I^2^* =75%, *P* < 0.01).

The heterogeneity may have been due to the use of repeated transcranial magnetic stimulation and other treatments. The results of the subgroup analysis showed that only the somatosensory interactive games group had significantly different ADL scores from those of the control group (Fig. 5).

### Quality of life in alzheimer's disease scale

**Fig. 6:**
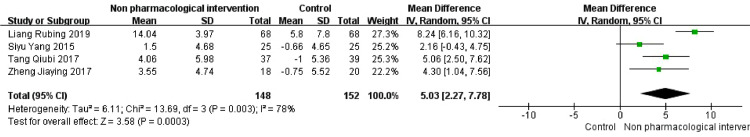
Forest plot of QoL-ADscores in older adults with Alzheimer's disease in the nonpharmacological intervention group versus control group.

A total of 148 older adults with Alzheimer's disease who underwent nonpharmacological interventions and 152 who did not undergo nonpharmacological interventions completed the QoL-AD scale before and after treatment. The random effects model was used for meta-analysis, and the results showed that the quality of life of the nonpharmacological intervention group was better than that of the control group [MD=5.03, 95% CI (2.27,7.78)] (Fig. 6). There was significant heterogeneity (*I^2^* =78%, *P* < 0.01).

### Montreal cognitive assessment

**Fig. 7:**
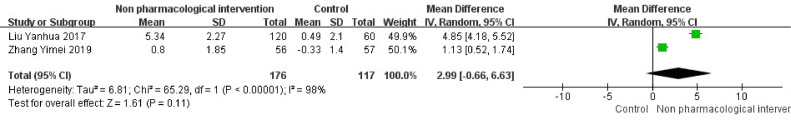
Forest plot of MoCA scores in older adults with Alzheimer's disease in the nonpharmacological intervention group versus control group.

A total of176 older adults with Alzheimer's disease who underwent nonpharmacological interventions and 117 who did not undergo nonpharmacological interventions completed the MoCA before and after treatment. The random effects model was used for meta-analysis, and the results showed that the difference in cognitive functioning between the nonpharmacological intervention group and the control group was not significant [MD=2.99, 95% CI (−0.66,6.63)] (Fig. 7). There was significant heterogeneity (*I^2^* =98%, *P* < 0.01).

### Neuropsychiatric inventory

**Fig. 8:**
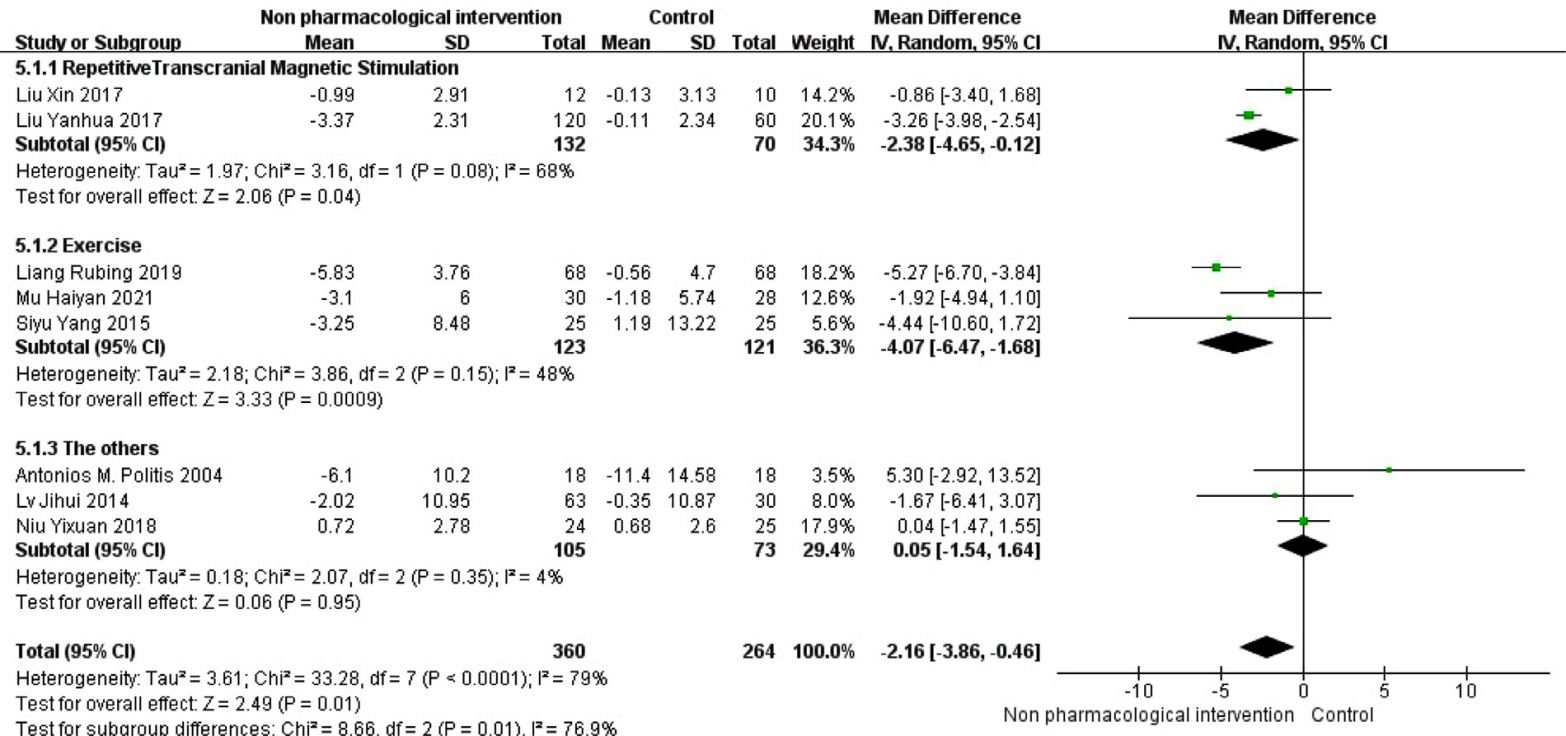
Forest plot of NPI scores in older adults with Alzheimer's disease in the nonpharmacological intervention group versus control group.

A total of 360 older adults with Alzheimer's disease who underwent nonpharmacological interventions and 264 who did not undergo nonpharmacological interventions completed the NPI before and after treatment. The random effects model was used for meta-analysis, and the results showed that the psychobehavioural symptoms of the nonpharmacological intervention group were less severe than those of the control group [MD=−2.16, 95% CI (−3.86,0.46)] (Fig. 8). There was significant heterogeneity (*I^2^* =79%, *P* < 0.01).

The heterogeneity may have been due to the rTMS treatment group. The results of the subgroup analysis showed that both the rTMS and exercise treatment groups had statistically significant differences in NPI scores compared with the control group (Fig. 8).

### Alzheimer's disease assessment scale-cognitive score

**Fig. 9:**
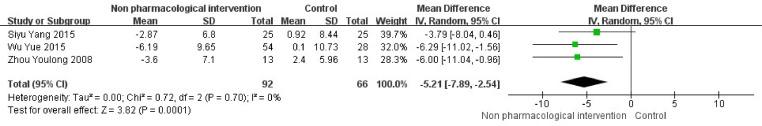
Forest plot of ADAS-cog scores in older adults with Alzheimer's disease in the nonpharmacological intervention group versus control group.

A total of 92 older adults with Alzheimer's disease who underwent nonpharmacological interventions and 66 who did not undergo nonpharmacological interventions completed the ADAS before and after treatment. The random effects model was used for meta-analysis, and the results showed that the cognitive functioning of the nonpharmacological intervention group was better than that of the control group [MD=−5.21, 95% CI (−7.89,2.54)] (Fig. 9). There was not significant heterogeneity (*I^2^* =0%, *P* = 0.70).

### Risk of bias

**Fig. 10:**
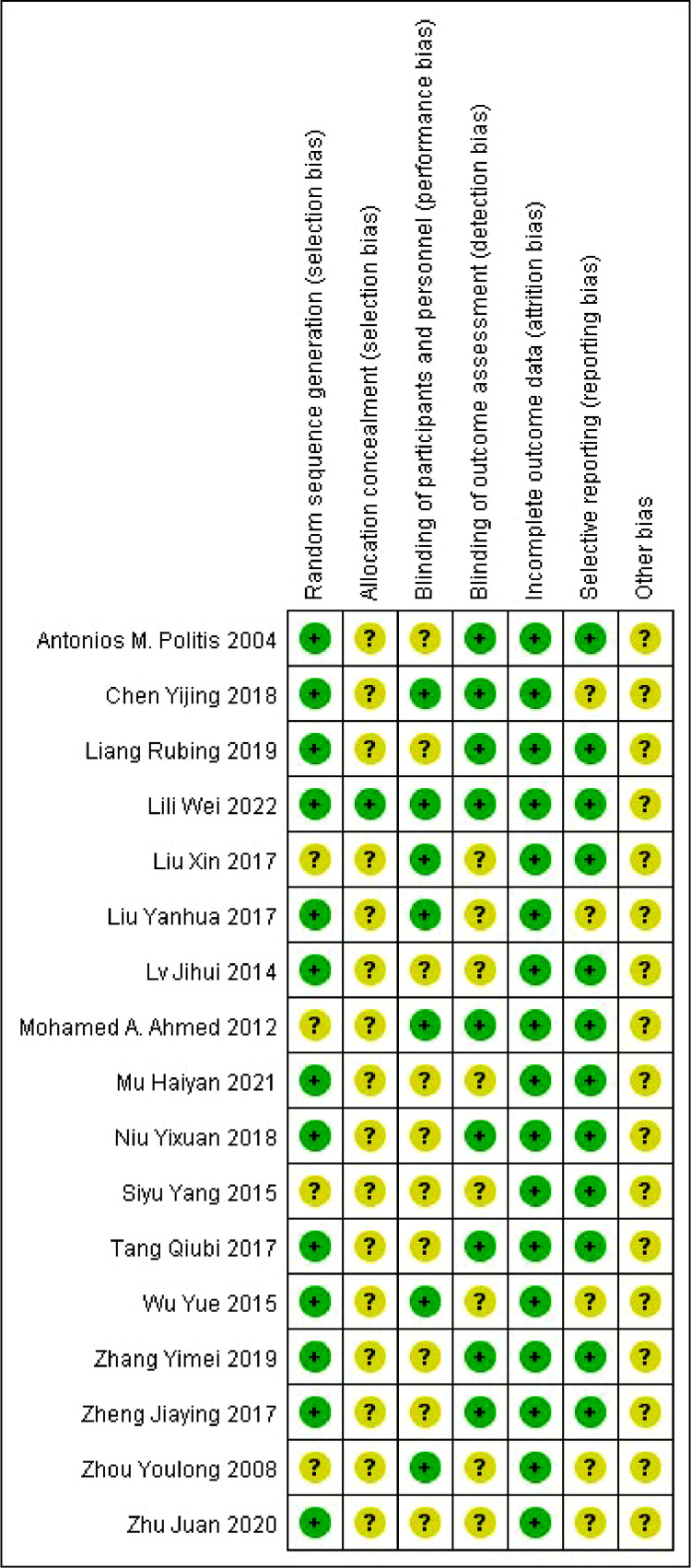
Risk of bias assessment results of included studies.

The results of the risk of bias assessment are shown in Fig. 10.

**Fig. 11:**
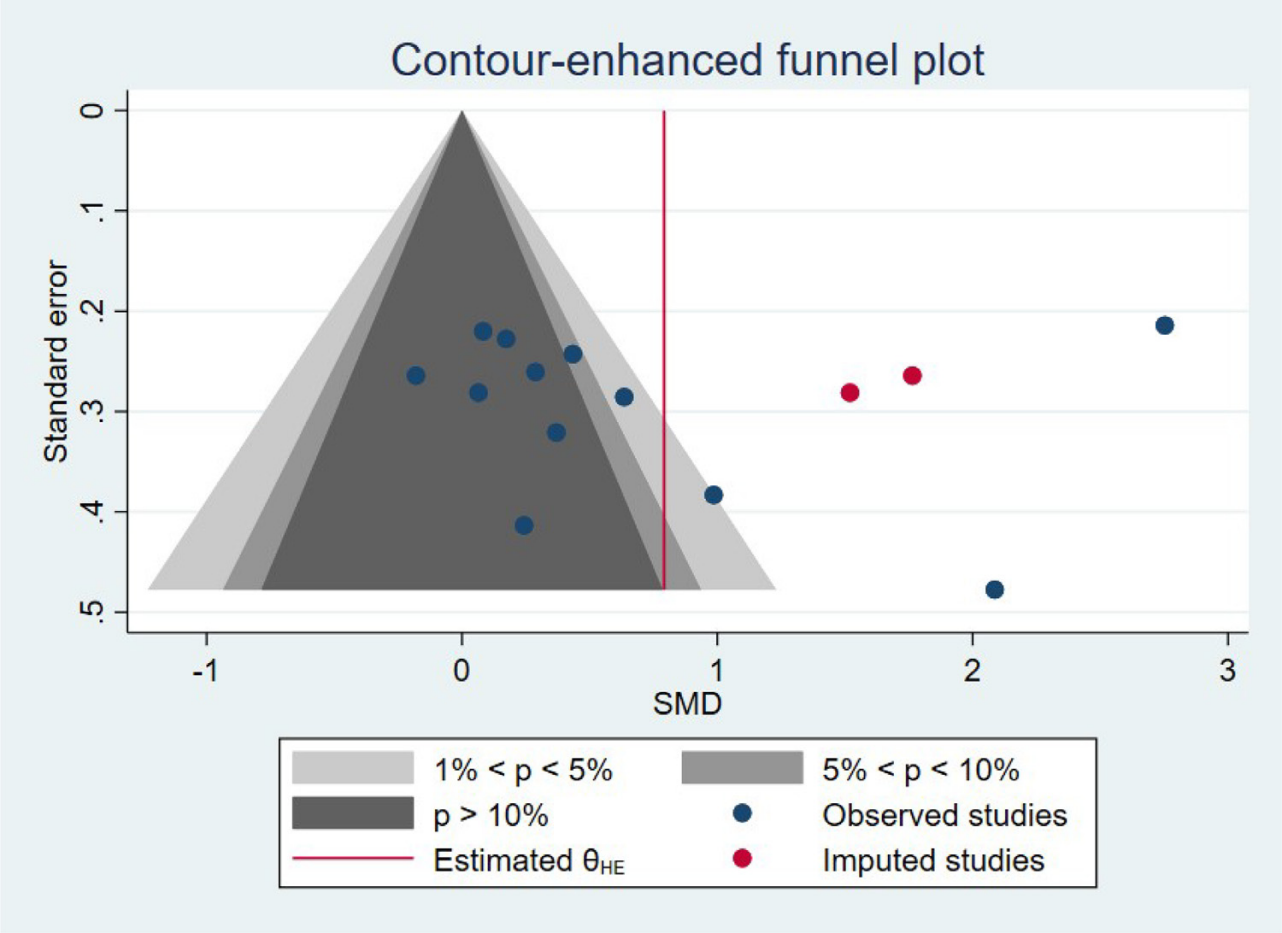
The clipping-contour-enhanced funnel plot for the scale scores of MMSE.

A clipping-contour-enhanced funnel plot was constructed for the MMSE scores to test for publication bias. The results showed that there was asymmetry in the funnel plot, suggesting that there may be a certain degree of publication bias (Fig. 11).

## Discussion

Overall, the nonpharmacological intervention was effective in improving cognitive functioning among older adults with mild-to-moderate Alzheimer's disease, as evidenced by the fact that, with the exception of the MoCA scale, the differences between the intervention group and the control group were significant for all the remaining scales. However, to date, the potential therapeutic effects of common nonpharmacologic therapies for AD and the associated mechanisms have not been fully elucidated. The hippocampus and prefrontal cortex are closely related to human cognitive function, and the possible mechanisms for improving cognitive functioning are that exercise interventions, cognitive interventions, and noninvasive brain stimulation in the prefrontal lobe promote prefrontal cortex activity and may also accelerate neural conduction and increase neural excitability by improving cortical plasticity and remodeling the connections between synapses.

Subgroup analyses of the different types of interventions revealed differences only in the NPI; there were no significant differences in the MMSE or ADL scores. One of the advantages of the NPI compared with the MMSE may be that the former is less influenced by cultural background. tACS enables non-invasive modulation of neural activity in the brain by applying weak currents at specific frequencies on the surface of the brain. Reinhart and his research team have recently found that tACS can effectively improve nine types of cognitive functions, including visual attention, working memory, long-term memory, executive control, fluid intelligence, learning, decision-making, motor learning, and motor memory [39]. This improvement is applicable not only to healthy subjects but also to elderly individuals and patients with neuropsychiatric disorders. Similarly, the potential treatment effects of tDCS, another form of electrical stimulation, and whether these effects have clinical significance, as well as how to optimally implement tDCS in a therapeutic setting, remain to be clarified [40]. rTMS is a painless and noninvasive neuromodulation technique in which strong magnetic pulses are transmitted through the scalp to the cortex to modulate cortical activity. Depending on the stimulation parameters, rTMS can either enhance or inhibit cortical excitability in target cortical areas [41]. Previous meta-analyses have also shown that compared to cognitively normal elderly individuals, patients with Alzheimer's disease exhibit significantly higher cortical excitability, lower cortical inhibition, and impaired cortical plasticity [18]. And it has been widely used to treat psychiatric disorders and improve cognitive function [42].

Similarly, compared to transcranial magnetic stimulation, subgroup analyses showed that the exercise intervention appeared to be the most effective, with significant differences in all three scales of the MMSE, ADL, and NPI compared to the control group. The hippocampus, as a cognitive memory storage region, often affects the cognitive memory of the body, and the hippocampus of AD patients often shows different degrees of atrophy [43]. Exercise can improve cognitive ability, and its specific mechanism may be attributed to an increase in hippocampal volume [44]. An increasing amount of research on the effects of exercise on AD has revealed that the exercise-induced reversal of cognitive decline may be related to several pathways: i. reducing inflammatory factor expression, balancing oxidative stress homeostasis inside and outside neuronal cells, and reducing neuronal cell damage [45]; ii. increasing vascular endothelial growth factor (VEGF) and decreasing vasopressor expression, promoting angiogenesis and enhancing cerebral blood flow; and iii. increasing the expression of brain-derived neurotrophic factor (BDNF) [46], promoting neural activity in the hippocampal region and improving synaptic plasticity [47]. Regular exercise can reduce morbidity among AD patients, but the optimal duration, intensity and frequency of exercise still need to be determined. Furthermore, when instructing patients to exercise, the cardiorespiratory function and exercise endurance of patients should be taken into account to prevent aggravation of the disease by exceeding the patient's tolerance.

In contrast, our subgroup analysis showed that music therapy did not seem to improve cognitive functioning in AD patients. This finding is inconsistent with previous research that has suggested that music promotes the lineage and solidification of memories, while the hippocampus and the dorsolateral prefrontal cortex show greater involvement under musical stimulation [48]. Music is inspirational, immersive, and emotional and has the ability to reawaken memories and emotions and alleviate anxiety and panic – these qualities lead to a synergistic effect that can alleviate many of the patient's symptoms. However, more high-quality randomized controlled trials may need to be included to resolve this discrepancy.

Other categories of nonpharmacological interventions, such as acupuncture, sensory training, increased social activity, and horticultural therapy, were not combined with any medication in the study; however, music therapy at home and abroad was even less common, and there were more cointerventions combined with medication, which does not allow us to confirm the effect of a single intervention.

In our study, nonpharmacological interventions have been shown to effectively improve cognitive function in elderly patients with Alzheimer's disease. These effective nonpharmacological interventions can provide family caregivers with the knowledge, skills, and support they need to address their caregiving challenges. In addition to enhancing the quality of life for patients, these interventions incur little to no additional costs compared to standard care, making them cost-effective from a societal perspective [49]. Our findings can offer insights for clinicians in treating Alzheimer's patients and also encourage healthcare payers to develop new insurance strategies for nonpharmacological interventions.

This study has several limitations. Although this study suggested that nonpharmacological therapies have certain advantages in improving cognitive functioning in AD patients, current clinical trials on nonpharmacological therapies for the treatment of AD still suffer from deficiencies such as small sample sizes, short duration of treatment, and lack of observation of sustained posttreatment effects, which are aspects that should be emphasized in future clinical studies. Furthermore, the current clinical assessments of AD-related cognitive function, daily living ability, and psychobehavioral symptoms are still based on scales, and future studies should observe the efficacy of treatment through more objective indicators. Due to the broad range of nonpharmacological interventions in real life, our search terms could not be fully comprehensive. And we did not search specialized psychological databases such as PsycINFO. In addition, there was a high degree of heterogeneity in the findings, and although we addressed this through the use of the random effects model and subgroup analyses, excluding individual articles with questionable data would have made the conclusions more stable and reliable.

## Data and materials availability

The datasets generated during the current study are available in the [raw data3] repository, [https://www.jianguoyun.com/#/sandbox/18c8fa4/7cd43bf96f7d135f/%2F/].

## Declaration of Generative AI and AI-assisted technologies in the writing process

I confirm that I have not used any AI at all.

## Funding

This work was supported by Changzhou Health Young Cultivation Project (CZQM2020101).

## CRediT authorship contribution statement

**Kejin Chen:** Investigation, Funding acquisition, Data curation. **Xiaoyan Zhao:** Writing – original draft, Conceptualization. **Jingwen Zhou:** Writing – original draft, Supervision, Resources, Formal analysis.

## Declaration of competing interest

The authors declare that they have no known competing financial interests or personal relationships that could have appeared to influence the work reported in this paper.
